# Avoid ‘prognostic paralysis’—just get ahead and plan and co-ordinate care

**DOI:** 10.1038/npjpcrm.2014.85

**Published:** 2014-10-09

**Authors:** Eleni Epiphaniou, Cathy Shipman, Richard Harding, Bruce Mason, Scott A Murray, Irene J Higginson, Barbara A Daveson

**Affiliations:** 1 Department of Palliative Care Policy and Rehabilitation, Cicely Saunders Institute, King’s College London, London, UK; 2 Primary Palliative Care Research Group, Centre for Population Health Sciences, University of Edinburgh, Edinburgh, UK

Dear Sirs,

We are grateful for the letters responding to our article which highlighted that poor last-year-of-life care for chronic obstructive pulmonary disease (COPD) patients was partly due to the prognostic uncertainty.^1^ In response, Crawford *et al*.^2^ called for more accurate prognostication as a way forward. This is fine in theory but near impossible in practice, and use of a greater number of tools may not be appropriate in fatigued and breathless patients. Instead we agree with the response by Kendall *et al.*^3^ that rather than wait to identify a transition point to trigger palliative care, we should avoid ‘prognostic paralysis’ and plan holistic care according to needs. This will encourage integrated, early use of palliative care alongside disease-oriented care. As palliative care input has been shown to improve care coordination, early integrated palliative care input should provide benefits for patients and their unpaid caregivers.^4^

One of the largest barriers to initiating generalist palliative care is the perception among patients, family carers and some health professionals that palliative care is a ‘death sentence’.^5^ Attempts to focus on the accuracy of prognostication will only reinforce that perception. Instead, we suggest that all patients with chronic illness such as COPD should have anticipatory care for expected deteriorations, which can be extended in scope as the illness progresses. In this way we can make progress towards the holistic, well-coordinated management of patients with one or often multiple chronic conditions by generalist clinicians in the community.^6^ This will improve the continuity of care and overall care coordination, as well as facilitate the access to specialist care as necessary, and should improve patient outcomes.
